# Gestational age and models for predicting gestational diabetes mellitus

**DOI:** 10.1007/s11306-025-02314-3

**Published:** 2025-09-26

**Authors:** Aisling Murphy, Jeffrey Gornbein, Ophelia Yin, Brian Koos

**Affiliations:** 1 University of California, Los Angeles, USA; 2 University of California, San Francisco, USA

**Keywords:** Metabolomics, Urine, Gestational diabetes mellitus

## Abstract

**Introduction:**

Gestational diabetes mellitus (GDM) is generally identified by measuring elevated maternal glycemic responses to an oral glucose load in late pregnancy (> 0.6 term). However, our preliminary study suggests that GDM could be identified with a high predictive accuracy (96%) in the first trimester (< 0.35 term) by characteristic changes in the metabolite profile of maternal urine. (Koos and Gornbein, American Journal of Obstetrics and Gynecology 224:215.e1–215.e7, 2021). The gestational decrease in insulin sensitivity and the accompanying perturbations of the maternal metabolome suggest that a distinguishing urinary metabolite algorithm could differ in later gestation.

**Objectives:**

This study was carried out (1) to identify the metabolites of late-pregnancy urine that are independently associated with GDM, (2) to select a metabolite subgroup for a predictive model for the disorder, (3) to compare the predictive accuracy of this late pregnancy algorithm with the model previously established for early pregnancy, and (4) to determine whether the late urinary markers of GDM likely contribute to the late pregnancy decline in insulin sensitivity.

**Methods:**

This observational nested case–control study comprised a cohort of 46 GDM patients matched with 46 control subjects (CON). Random urine samples were collected at ≥ 24 weeks’ gestation and were analyzed by a global metabolomics platform. A consensus of three multivariate criteria was used to distinguish GDM from CON subjects, and a classification tree of selected metabolites was utilized to compute a model that separated GDM vs CON.

**Results:**

The GDM and CON groups were similar with respect to maternal age, pre-pregnancy BMI and gestational age at urine collection [GDM 30.8 $\pm$ 3.6(SD); CON [30.5 ± 3.6] weeks as they were matched by these variables. Three multivariate criteria identified eight metabolites simultaneously separating GDM from CON subjects, comprising five markers of mitochondrial dysfunction and three of inflammation/oxidative stress. A five-level classification tree incorporating four of the eight metabolites predicted GDM with an unweighted accuracy of 89%. The model derived from early pregnancy urine also had a high predictive accuracy (85.9%).

**Conclusion:**

The late pregnancy urine metabolites independently linked to GDM were markers for diminished insulin sensitivity and glucose-stimulated insulin release. The high predictive accuracy of the models in both early and late pregnancy in this cohort supports the notion that a urinary metabolite phenotype may separate GDM vs CON across both early and late gestation. A large validation study should be conducted to affirm the accuracy of this noninvasive and time-efficient technology in identifying GDM.

## Introduction

Gestational diabetes mellitus (GDM) is a common pregnancy complication that signals major risks for future cardiometabolic morbidity in both mother and child. (Kautzky-Willer et al., 2023; Koos & Gornbein, 2021; Rudge et al., 2023; Sweeting et al., 2022; Wicklow & Retnakaran, 2023; Xie et al., 2022; Ye et al., 2022).

For over 70 years, oral glucose challenges have been used to identify GDM, which is now generally recognized in late gestation by a 2-h, 75 g or 3-h, 100 g oral glucose tolerance test (GTT). While there is little consensus on the diagnostic utility of the 75 g versus 100 g GTT, (ACOG Practice Bulletin no.^190^., 2018; Huhn et al., 2018; Mallik & Huda, 2023; Tehrani et al., 2022; Tsakiridis et al., 2021; US Preventive Services Task Force., 2021) both approaches are onerous due to inconvenient testing, time involved, fasting requirement, and the emotional and physical trauma of repeated venipuncture. Variable processing of blood samples also impairs the accuracy and interpretation of glucose measurements. (Jamieson et al., 2023; Potter et al., 2020) These deficiencies highlight the exigency for a more amenable and reproducible diagnostic tool.

GDM is a heterogenous, multifactorial disorder involving genetic and environmental modulators of insulin production and sensitivity. (Benhalima et al., 2019; Kotzaeridi et al., 2021; Napso et al., 2018; Selen et al., 2022; Sharma et al., 2022; Wang et al., 2023) Metabolomics studies have revealed metabolite perturbations in maternal urine and blood that are associated with GDM and are thus potential identifiers of the disorder. (Alesi et al., 2021; Chen et al., 2018; Hou et al., 2018; Lopez-Hernandez et al., 2019; Lu & Hu, 2022; Mao et al., 2017; Tian et al., 2021; Walejko et al., 2020; Zhao et al., 2019) However, predictive algorithms involving these markers have lacked sufficient diagnostic accuracy in separating GDM versus non-GDM subjects.

**Fig. 1:**
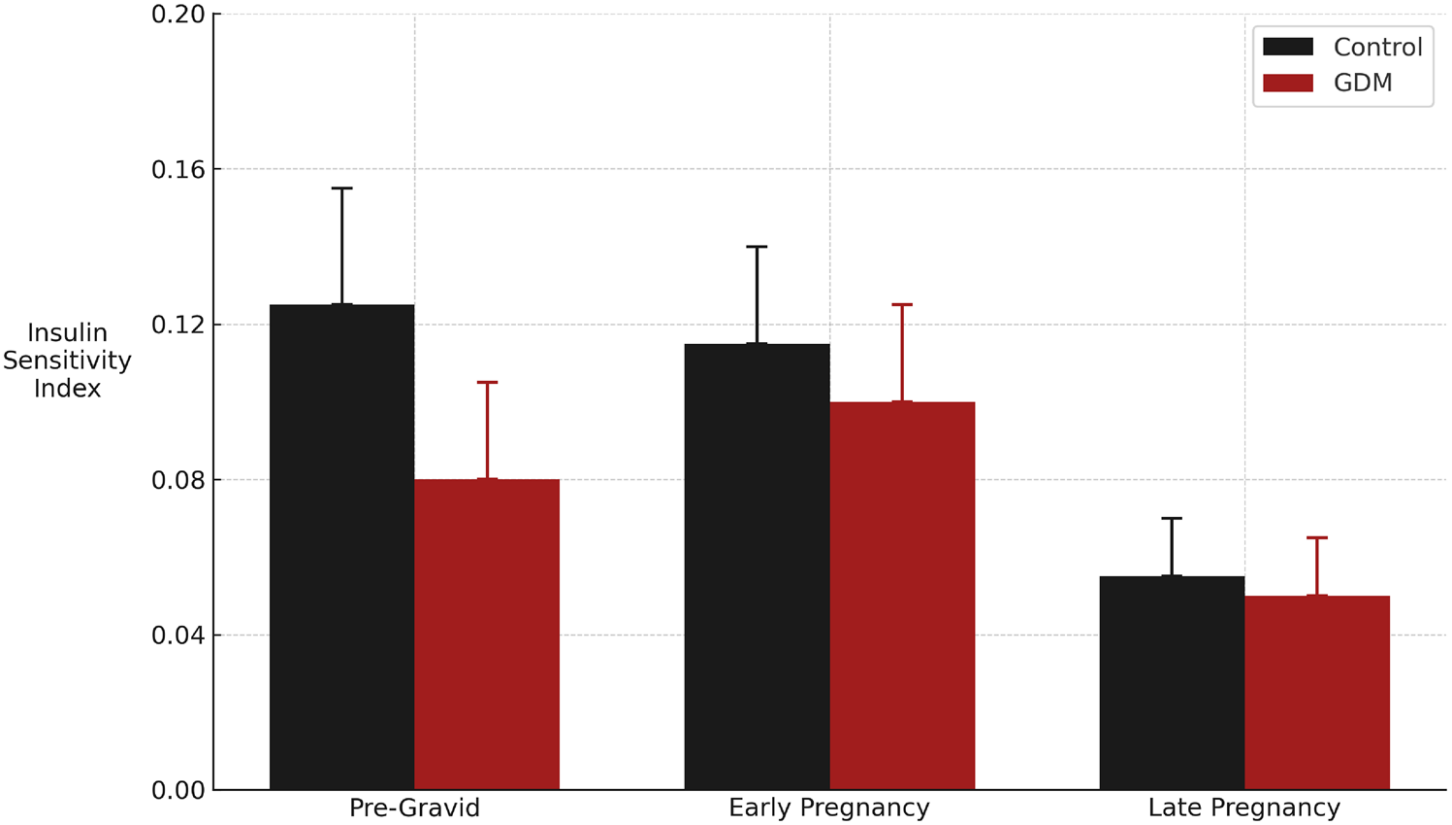
Longitudinal changes in insulin sensitivity index (mean ± SD). Longitudinal changes in insulin sensitivity in n = 6 control and n = 10 GDM subjects demonstrating a significant (*P* = 0.0001) decrease in insulin sensitivity index in both groups with advancing gestation. (Adapted from Catalano et al., 1993)

Progress in high-throughput metabolomics, statistical analysis, and machine learning in preliminary studies have enabled the computation of a metabolite model from early pregnancy urine that had a high predictive accuracy (unweighted mean of sensitivity and specificity) of 96.7%. (Koos & Gornbein, 2021) The maternal metabolome changes dynamically and profoundly over the course of gestation with significant changes occurring in over 70% of 48 interrogated metabolic pathways. (Liang et al., 2020) These adjustments in the molecular milieu also perturb the complex interaction of metabolic pathways. In GDM, the alterations in maternal metabolism that emerge during the second trimester can blunt insulin signaling and sensitivity (Fig. 1), which leads to increased insulin resistance and glucose intolerance. (Yang et al., 2018) Thus, the metabolites in late pregnancy that predict GDM may differ from those identified in the first trimester.

This study involves the metabolomics analysis of late-pregnancy urine from the cohort of gravidas who comprised our previous study in early pregnancy. (Koos & Gornbein, 2021) The objectives are to (1) characterize the metabolite phenotype of late-pregnancy urine that is independently associated with GDM, (2) select a subgroup of these metabolites for a predictive model of GDM, (3) compare the accuracy of this late-pregnancy algorithm with that derived from metabolites of the early-pregnancy model.

## Methods

### Study design

This observational nested case–control study involved the non-targeted metabolomics identification of small molecules in maternal urine collected at ≥ 24 weeks’ gestation. The Global Alliance to Prevent Prematurity and Stillbirth (GAPPS; Seattle, WA) supplied the de-identified, randomly collected, and frozen (−80 °C) urine samples and demographic information of patients recruited from antenatal clinics in Washington State. All participating subjects signed written informed consent forms approved by institutional review boards at the University of Washington Medical Center; Swedish Medical Center, Seattle; Yakima Memorial Hospital; and Children’s Hospital Seattle. (Koos & Gornbein, 2021).

### Subjects

A cohort of healthy singleton gravidas at ≥ 24 weeks’ gestation provided randomly collected urine samples and underwent GDM screening at ≥ 24 weeks’ gestation. GDM was diagnosed by institutional criteria using 1-h, 50 g GCT, a 3-h 100 g GTT or HbA1c. GDM and CON gravidas were matched by age, pre-pregnancy BMI, and gestational age (GA) at urine collection. A dissimilarity score equation (Mahalanobis, 1936) determined the similarity of the match:

$$ \begin{aligned} {\text{Score}} = & \;\left( {3\left[ {\Delta {\text{GA1}}/{\text{SD}}_{{{\text{GA1}}}} + \;\Delta {\text{GA2}}/{\text{SD}}_{{{\text{GA2}}}} } \right)/2} \right] \\ & + 2_{*} {\text{Age}}/{\text{SD}}_{{{\text{age}}}} + 1\Delta {\text{BMI}}/{\text{SD}}_{{{\text{BMI}}}} )/6 \\ \end{aligned} $$

where ∆ is the absolute difference between a given case and the respective CON for GA1 (urine collected in early gestation), GA2 (urine collected ≥ 24 weeks’ pregnancy), maternal age, or pre-pregnancy BMI. SD represents the pooled SD of GA1, GA2, age, or BMI with the score in SD (Z) units. The score is a special case of the Mahalanobis distance (Mahalanobis, 1936) in SD units where the covariances were conservatively set to zero. The score was calculated for all combinations of the 46 cases with a larger pool of potential controls. The 46 normal gravidas with the smallest scores (best match) were selected. Both GA1 and GA2 were used in matching, but only data from GA2 were involved in the analysis.

### Metabolomics analysis

Biochemicals (MW < 1000) were identified by UPLC-MS/MS with both positive and negative ion mode electron-spray ionization and gas chromatography (Metabolon, Inc., Durham, NC). Log transformed peak areas of metabolites were scaled to the respective median peak area with adjustments for fluid intake.

### Statistical analysis

Biochemicals endogenous to maternal metabolism were used in the analyses. Xenobiotics and partially characterized molecules were excluded. There were 626 candidate biochemicals.

#### Bivariate analysis

The values for comparing normally distributed continuous variables between groups were computed using t-tests, while those not normally distributed were calculated by the Wilcoxon rank sum test. The *P* values for comparing categorical variables (race, ethnicity) were computed by the Fisher exact test. Log scale metabolites for GDM and CON were summarized as means ± SDs with differences nominally significant at *P* < 0.05 since the metabolites had a normal distribution on the log scale.

#### Multivariate analysis

Both random forest and gradient boosting models (GBM) were used to choose a subset of the 626 candidate metabolites to distinguish between GDM and controls. The relevant importance of each metabolite was assessed via random forest predictive accuracy, random forest Gini worsening, and gradient boosting relative influence. A consensus ranking using all three of these criteria was used to select a final subset of top-ranked endogenous compounds.

A classification tree using the top-ranked metabolites by the three multivariate criteria was constructed for the final predictive model. Sensitivity, specificity, accuracy (unweighted mean of specificity and sensitivity), and the receiver-operating characteristic area under the curve (ROC_AUC_) were computed to evaluate the accuracy of the final model.

Computations were performed using SAS (version 9.4; SAS Institute Inc, Cary, N.C) and R (version 3.5.2; R Project for Statistical Computing, https://www.r-project.org

## Results

### Case–control characteristics

The mean dissimilarity score of 0.37 ± 0.20 units confirmed that the GDM-CON pairs were well-matched for mean maternal age (GDM 32 ± 4.7, CON 31.8 ± 4.2 years; *P* = 0.56), pre-pregnancy BMI (GDM 31.5 ± 6.8, CON 29.9 ± 6.3 kg/m^2^; *P* = 0.18), and GA at urine collection (GDM 30.8 ± 3.6, CON 30.5 ± 3.0; *P* = 0.36). No significant differences were observed for the unmatched parity and race/ethnicity (partity mean ± SD; 2.5 ± 1.3, GDM and 1.9 ± 1.0, CON, *P* = 0.31).

### GDM diagnosis

GDM was diagnosed in the 46 cases based on an abnormal GTT in 22 gravidas (47.8%), GCT (> 200 mg/dl) in 6 (13%), GCT (171–187 mg/dl) in 15 (32.6%), fasting blood glucose > 95 mg/dl in one (2.2%) and a first trimester HbA1c ≥ 5.7% in 2 (4.3%). GDM was diagnosed in 31 (67%) subjects ≥ 24 weeks’ gestation and eight (17.4%) with risk factors < 12 weeks’ gestation. Maternal glycemia was controlled by diet (47.8%), oral hypoglycemic agents (41.2%), and insulin (10.8%).

### Bivariate analysis

One-metabolite-at-a-time analysis was used to determine the accuracy of the 626 individual endogenous metabolites distinguishing GDM. The top 10 of the 626 metabolites had individual predictive accuracies ranging from 66.3 to 69.6%. The three highest ranked metabolites were 3-hydroxybutyrate, 3-methylhexanolycarnitine, and saccharopine.

### Multivariate analysis

Individual scree plots of the three multivariate criteria ranked metabolites that independently separated GDM from CON. Thirty of the 626 endogenous metabolites comprised the top 30 by at least one of the three criteria, while 13 of the 30 compounds were highly ranked by at least two. All three criteria were used to select the final eight candidates for the late pregnancy model (Table 1). The eight metabolites selected by the three multivariate criteria were among the 11 highest ranked by bivariate analysis.

**Table 1:** Candidate urinary metabolites by consensus multivariate criteria for distinguishing GDM vs CON

Metabolite	Functional Pathway	Effect size^a^
3-Hydroxybutyrate^b,c^	Fatty acid catabolism	0.815
Homocarnosine^b,c^	GABA metabolism antioxidant	−0.663
1,5-anhydroglucitol^b,c^	Glucose competitor for renal tubular transport	−0.383
3-Hydroxydodecanedioate^b,c^	Medium chain fatty acid catabolism	0.759
(R)3-Hydroxybutyrylcarnitine^c^	Fatty acid catabolism	0.628
3-Methylhexanolylcarnitine^c^	Fatty acid metabolism	0.673
Saccharopine^c^	Lysine catabolism	0.822
Acetylcholine^c^	Choline metabolism neurotransmitter	0.704

^a^(GDM-CON)/SD, log scale; ^b^selected for final predictive model; ^c^nominal *P* < 0.05

### Classification tree

**Fig. 2:**
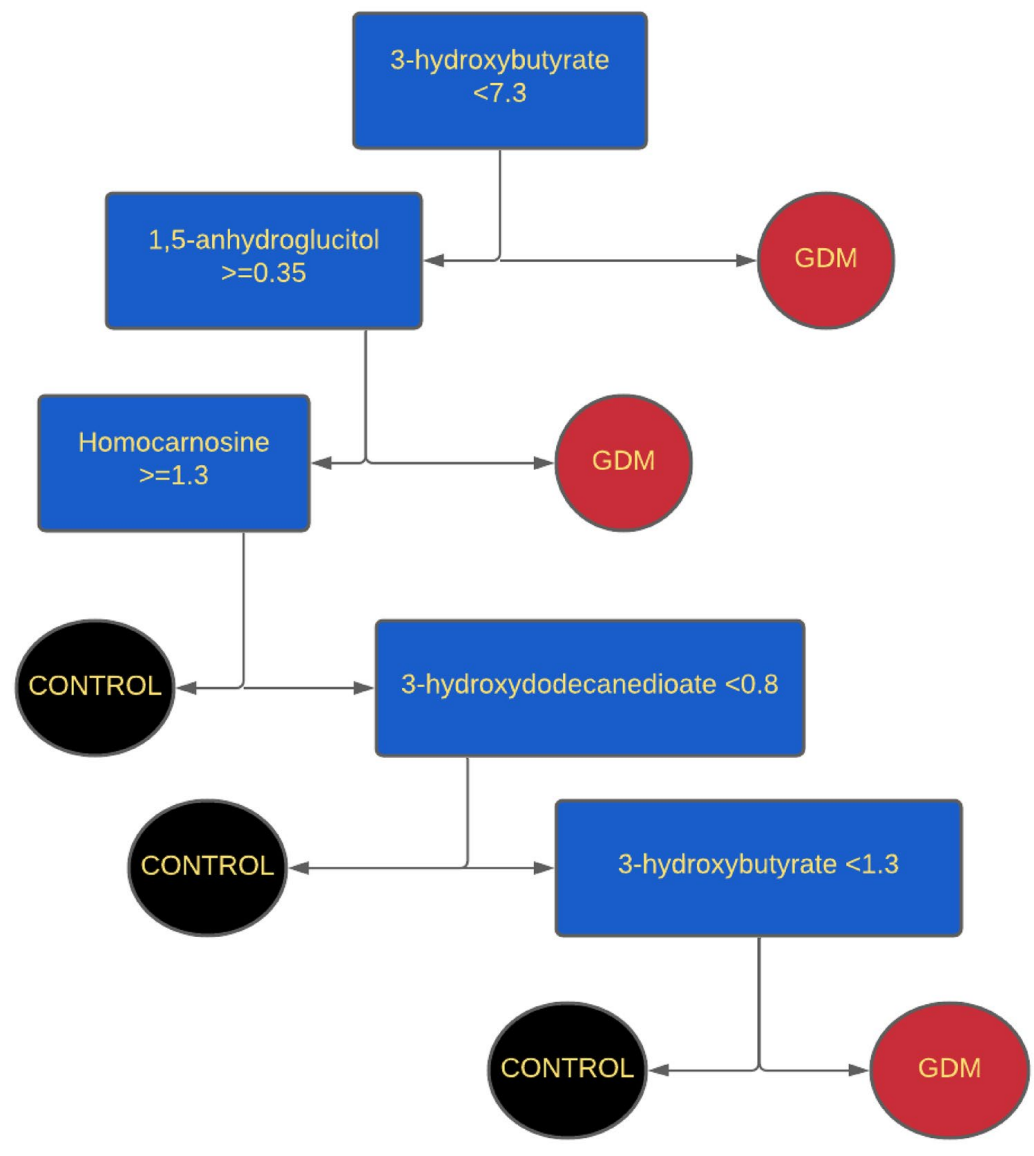
Classification tree model using late-pregnancy urine metabolites. This final model depicts the classification tree analysis of the four metabolites of late pregnancy urine with the best prediction accuracy of 89.1% for separating GDM from non-GDM. The metabolite concentration index for a metabolite at each tree node (e.g., 3-hydroxybutyrate < 7.3) determines whether the tree is traversed to the left if the condition is true (+ condition) or to the right if it is false (- condition). The metabolite level is scaled to the respective median and corrected for osmolality in the original scale

A classification tree analysis of the eight identified compounds selected four metabolites for the final predictive algorithm. The five-level classification tree identified GDM with an accuracy of 89.1% (Table 2, Fig. 2).

**Table 2:** Sensitivity, specificity, accuracy, and ROC area for markers simultaneously distinguishing GDM vs non-GDM in late pregnancy

Tree Metabolites	Sensitivity (%)	Specificity (%)	Accuracy (%)	ROC area
Late pregnancy metabolites*	87.0	91.3	89.1	0.923
3-hydroxybutyrate				
1,5-anhydroglucitol				
Homocarnosine				
3-hydroxydodecanedioate				
Early pregnancy metabolites**	87.0	84.8	85.9	0.880
Dihydroorotate				
Phenol glucuronide				
Nicotinate ribonucleoside				
Saccharopine				

*31 ± 3.3 weeks’ gestation; **12 ± 2.9 weeks’ gestation (10)

Two algorithms were derived from consensus metabolites derived from (1) late pregnancy urine, and (2) early pregnancy urine

**Fig. 3:**
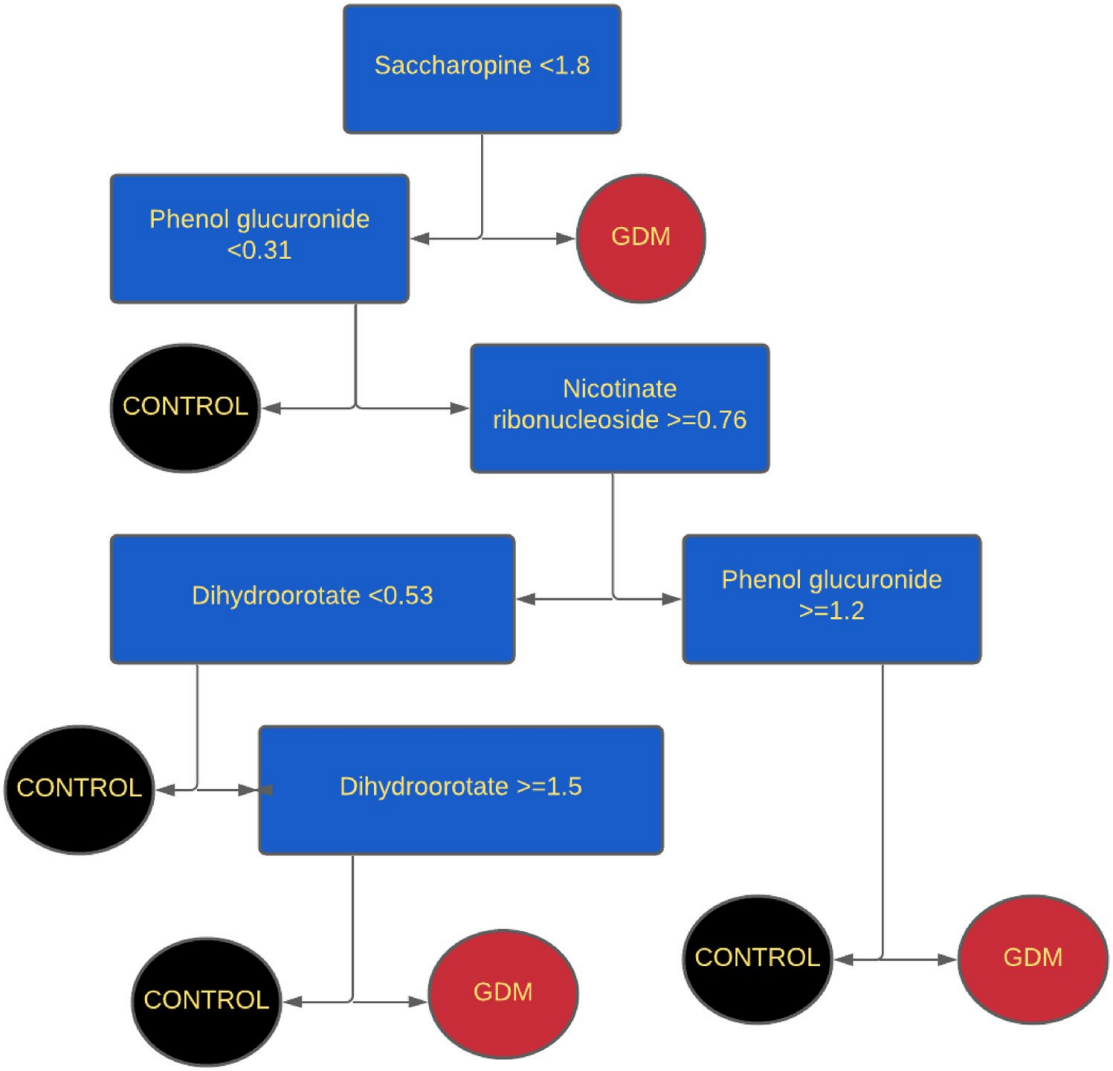
Classification tree for late pregnancy using model metabolites of early pregnancy. This model shows the classification tree analysis using 4 of the 7 metabolites identified previously as highly accurate (96.7%) in distinguishing GDM from non-GDM in early pregnancy. This model had a prediction accuracy of 85.9% for late-pregnancy urine. The condition displayed at each node represents the metabolite level scaled to the respective median and corrected for osmolality in the ordinal scale

A second classification tree was created using the seven metabolites of algorithm previously derived from early pregnancy urine (Table 3). This tree comprised only four of the seven metabolites of the first trimester model (Fig. 3). Including the remaining three (lanthionine, argininate, 7,8-dihydroneophterin) did not improve the predictive accuracy of 85.9%.

**Table 3:** Candidate urinary metabolites for late second-trimester samples compared to those reported for first trimester

Functional Pathway	First Trimester	Second Trimester
Amino acid metabolism	**Arginate (0.733)**^**†**^	
	**Saccharopine** (0.648)^†^	Saccharopine (0.822)
	Dopamine (0.254)	
Fatty acid metabolism	Octanoylcarnitine (0.124)	**3-hydroxybutyrate (0.815)**
	Methylglutarate (0.108)	**3-hydroxydodecanedioate (0.759)**
		3-hydroxybutyrylcarnitine (0.628*)*
		3-methylhenanoylcarnitine (0.673)
Tricarboxylic acid cycle	Isocitrate lactone (0.408)	
Oxidative stress/inflammation	**Lanthionine (-0.509)**	**Homocarnosine (-0.663)**
	**7,8-dihydroneopterin (0.464)**^**†**^	Acetylcholine (0.704)
	**Nicotinate ribonucleoside (0.655)**^**†**^	
Pyrimidine synthesis	**Dihydroorotate (0.558)**	
Phenol metabolism**	**phenol glucuronide (0.596)**^**†**^	
Renal glucose excretion		1,5-anhydroglucitol (-0.383)***

(Koos & Gornbein, 2021) Metabolites in bold type were selected for each of the final two models

(n) = effect size [GDM-CON)/SD, log scale]; **gut microbiota; ***competitive inhibitor of renal glucose excretion; ^†^nominal *P* < *0.05*

## Discussion

Maternal insulin sensitivity before 14 weeks’ gestation is the same or slightly higher compared to preconception values. Thereafter, insulin sensitivity progressively declines in later pregnancy, as shown in Fig. 1. This gestational decline in insulin sensitivity arises from increased maternal adiposity, placental growth hormone, and other factors. (Catalano, 2014; Catalano et al., 1991; Kampmann et al., 2019; Usman et al., 2023) The resulting changes in available substrates and finer tuned controllers determine the composition of the fuel (glucose versus fatty acids) driving mitochondrial energy production. (Gonzalez-Rellan et al., 2023; Hue & Taegtmeyer, 2009; Jeon, 2016; Pappas et al., 2023; Randle et al., 1963; Wasserman, 2022).

**Fig. 4:**
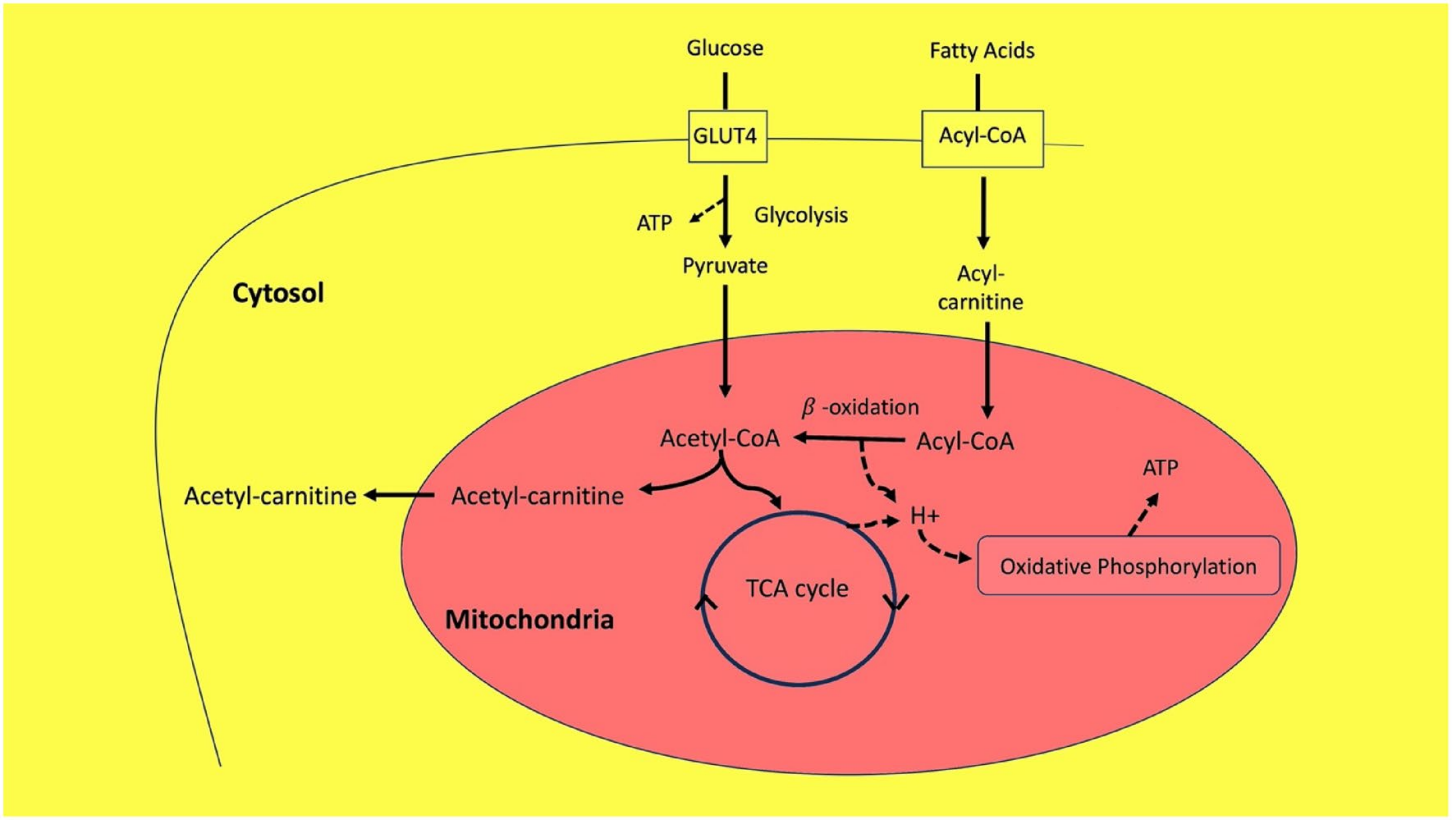
Outline of mitochondrial energy metabolism. In skeletal and cardiac muscle, both glucose and fatty acids can both be utilized for mitochondrial ATP production. Glucose traverses the plasma membrane of myocytes via GLUT4, an equilibrating glucose transporter. The subsequent phosphorylation of glucose by hexokinase II determines the net glucose uptake by muscle tissue, the major site of systemic insulin sensitivity.^41^ Insulin regulates both GLUT4 and hexokinase II activity, and pathologic perturbations of either are potentially involved in insulin resistance. Glycolysis yields pyruvate, which forms acetyl -CoA for the TCA cycle that drives drive ATP production by the electron transfer chain. In oxygen-deficiency (anaerobic metabolism) or mitochondrial dysfunction, pyruvate is converted to lactate, which along with degradation of muscle glycogen and phosphocreatine, contributes to ATP synthesis. At the cellular membrane, fatty acids combine with CoA to form acyl-CoAs, which are converted to acyl-carnitines that cross the inner mitochondrial membrane. Acyl-carnitine is subsequently converted to acetyl-CoA for β-oxidation. In this process, acyl-CoA is catabolized sequentially in two-carbon segments to form acetyl-CoA. For example, an eight-carbon acyl chain results in four molecules of acetyl-CoA, which, via the TCA cycle, drives ATP production by the electron transfer chain. In type 2 diabetes (T2D), high levels of acetyl-CoA can saturate the TCA cycle, and the excess utilized via an alternate pathway.^45^ For example, glycolysis generates pyruvate, which is converted to acetyl-coenzyme A (CoA). Acetyl-CoA supports the tricarboxylic acid (TCA) cycle, which, in turn, provides the energy that drives mitochondrial ATP production. In contrast, fatty acids are transported to the mitochondria where sequential β-oxidation of 2C units of acyl-CoAs yields one acetyl-CoA and one ATP per cycle. (Batchuluum et al., 2018; Houten et al., 2016; Lopaschuk, 2016)

Glucose and fatty acids differ in the metabolic pathways involved in energy production (Fig. 4).

The increased insulin resistance displayed during the second and third trimesters results in a greater contribution of fatty acids to maternal mitochondrial ATP production. By reducing maternal utilization of glucose, this metabolic adaptation promotes placental transfer of glucose from mother to the fetus, which provides critical substrate support for fetal metabolism and growth.

The maternal pancreatic β-cells normally increase insulin secretion to counter the rise in insulin resistance after 14 weeks’ gestation. In GDM, this β-cell compensation is insufficient to counter the gestational fall in insulin sensitivity. (Usman et al., 2023) The ensuing inefficiency in maternal glucose utilization increases maternal (1) insulin requirements, (2) energy dependence on fatty acid substrates, and (3) circulating blood glucose levels.

T2D is associated with an impairment of L-carnitine and acyl-carnitine entry into mitochondria. The resulting elevation in circulating levels signal a dysfunction in mitochondrial oxidation of fatty acids. (Batchuluum et al., 2018; Reuter & Evans, 2012).

Both early-onset T2D and GDM are associated with higher circulating concentrations of medium-chain (C6-C12) acyl-carnitines. These lipid metabolites diminish both (1) insulin sensitivity and (2) the glucose-stimulated insulin release by pancreatic β-cells and thus contribute to the dysregulation of maternal glycemia after mid-gestation. (Batchuluum et al., 2018; Roy et al., 2018).

The current study revealed that in late gestation, half of the eight consensus metabolites independently linked to GDM involved disruptions in fatty acid catabolism. These included a greater urinary excretion of one ketone (3-hydroxybutyrate), one small chain (< 6C) acylcarnitine ester (3-hydroxy-butyrylcarnitine), one medium-chain (6–12C) acylcarnitine (3-methylhexanolylcarnitine), and one medium chain fatty acid metabolite (3-hydroxydodecanedioate). Not surprisingly, fatty acid derivatives were dominant constituents of the predictive model.

### Amino acid metabolism

Lysine catabolism generates saccharopine and 2-aminoadipate. (Scislowski et al., 1994) In nonpregnant subjects elevated fasting levels of plasma 2-aminoadipic acid raised the long-term risk of T2D, (Wang et al., 2013) and in late pregnancy plasma lysine levels correlated positively with GDM. (Park et al., 2015) In the present study urine saccharopine excretion was simultaneously positively associated with GDM, as observed for first-trimester urine. (Koos & Gornbein, 2021) (Table 2) The elevated urinary excretion of saccharopine is consistent with a GDM-associated rise in hepatic and/or renal metabolism of lysine. (Gatrell et al., 2013).

### Inflammation and oxidation

Hyperglycemia and elevated free fatty acids trigger the release of pro-inflammatory mediators and oxidative stressors, which, in turn, promote insulin resistance and impair insulin release by pancreatic β-cells. (Lee et al., 2018; Perry et al., 2015; Pinto et al., 2023; Plows et al., 2018; Usman et al., 2023).

Cohort studies indicate that GDM subjects express proinflammatory perturbations in the first trimester, such as increased plasma cytokines, gut microbial dysbiosis, (Pinto et al., 2023) and characteristic metabolite perturbations in urine. (Koos & Gornbein, 2021) These observations are consistent with the involvement of proinflammatory mediators in early and late gestation. (Kirwan et al., 2002; Piao et al., 2019; Ye et al., 2023).

GABA is expressed in the brain and other tissues, including pancreatic beta cells. (Tillakaratne et al., 1995; Vincent et al., 1983) GABA combines with histidine to form the dipeptide homocarnosine, an antioxidant, anti-inflammatory compound, and an inhibitory neurotransmitter. The inclusion of homocarnosine in the algorithm is consistent with the GDM-associated metabolic disruptions related to increased oxidative stress/inflammation. (Huang et al., 2018; Peters et al., 2015; Teufel et al., 2003).

Plasma acetylcholine derives largely from non-neuronal tissues, including the placenta. (King et al., 1991; McLatchie et al., 2021; Wessler et al., 2001, 2003) In the current study, urine acetylcholine excretion was positively and independently associated with GDM. Circulating acetylcholine is a marker of inflammation, (Cox et al., 2020) and the raised renal excretion in GDM subjects in late pregnancy is consistent with heightened placental inflammation in this disorder. (Pen et al., 2021).

### Renal glucose excretion

Removing one hydroxyl group from glucose results in 5-anhydroglucitol, a diet-sourced compound that is insignificantly metabolized by human tissues. (Dworacka et al., 2006; Kim & Park, 2013; Pramodkumar et al., 2019) Blood levels of 5-anhydroglucitol are independent of prandial state, body weight or age. Because of renal tubular reabsorption, little of this compound undergoes urinary elimination.

Glucose competes with 5-anhydroglucitol for the renal tubular transporter. In type 2 DM the augmented glucose delivery to the renal tubules reduces renal tubular uptake of 5-anhydroglucitol and thereby (1) increases urinary excretion and (2) reduces circulating levels. Low serum levels of 1–5-anhydroglucitol are markers for intermediate- or short-term hyperglycemia in type 2 DM and are a modest predictor (ROC_AUC_ ~0.69%) of GDM. (Ma et al., 2017).

### Comparison accuracy of prediction model

The computed algorithm derived from urine metabolites independently linked to GDM in late pregnancy had a high accuracy in separating GDM versus CON (ROC_AU_ 0.923; accuracy 89.1%). The metabolite model previously derived from early pregnancy urine similarly had a high discriminatory power (ROC_AU_ 0.88, accuracy 83.9%).

## Conclusion

### Clinical implications

In GDM subjects proinflammatory markers are expressed in the urine, plasma, and gut microbiota during early pregnancy. (Koos & Gornbein, 2021; Pinto et al., 2023; Piras et al., 2022) These observations are consistent with GDM subjects having dysbiosis of the microbiome in early pregnancy that contributes to the pathogenesis of disorder. The high accuracy of the computed predictive models in both early and late pregnancy in this cohort suggests that a metabolite phenotype in maternal urine could accurately detect GDM across gestation.

### Research implications

Alterations consistent with inflammatory processes have been reported for urine, plasma, and feces weeks before the second trimester fall in insulin sensitivity. (Bankole et al., 2022; Dias et al., 2023; Koos & Gornbein, 2021; Law et al., 2017; Pinto et al., 2023; Piras et al., 2022; Teixeira et al., 2023) Thus, metabolite markers of inflammation expressed during early gestation, even before conception, may signal inflammatory contributors to the pathogenesis of GDM. Longitudinal studies should be conducted to: (1) determine the source(s) and cause of the inflammation, (2) identify potential preconception markers, and (3) assess the accuracy of metabolite models for predicting GDM.

### Strengths and limitations

The strengths of this study include the nested, longitudinal design, which facilitated comparisons between early and late pregnancy. Another advantage is the large number of untargeted metabolites with measurements corrected for maternal hydration. Other strengths include the use of consensus multivariate (GBM, random forest) and non-logistic candidate variable screening which did not require linearity or additivity when selecting a smaller array of candidate metabolites from which to derive the final candidates for the tree model. The candidate metabolites of the model were functionally related to the putative pathophysiology of insulin resistance and glucose intolerance, which supports the metabolite selections for the model.

This investigation is limited by the relatively small size and variable diagnostic identifiers, as well as the lack of an external validation dataset. Thus, it is critical that the results should be validated by a much larger study with more uniform criteria for the disorder.

## Summary

Urine metabolites independently associated with GDM are expressed before the second trimester rise in insulin resistance. In both early and late pregnancy, the included molecules involved in the putative root cause (oxidative stress/inflammation) of insulin resistance and impaired insulin secretion by pancreatic β-cells. The selected metabolites (e.g., medium chain fatty acids, 5-anhydroglucitol) of late pregnancy urine revealed mitochondrial dysfunction, which signaled a further impairment of glucose tolerance. Metabolite models computed from early- or late-pregnancy urine had a high accuracy in identifying GDM subjects. The results provide further optimism for the development of an early, non-invasive test for GDM. These promising observations should be confirmed by a large validation study.

## Data Availability

Data is provided within the manuscript.
